# Small Extracellular Vesicles and COVID19—Using the “Trojan Horse” to Tackle the Giant

**DOI:** 10.3390/cells10123383

**Published:** 2021-12-01

**Authors:** Blanka Maria Borowiec, Ana Angelova Volponi, Paul Mozdziak, Bartosz Kempisty, Marta Dyszkiewicz-Konwińska

**Affiliations:** 1 Department of Histology and Embryology, Poznan University of Medical Sciences, 60-781 Poznan, Poland; blanka.borowiec@student.ump.edu.pl (B.M.B.); bkempisty@ump.edu.pl (B.K.); 2 Centre for Craniofacial and Regenerative Biology, Faculty for Dentistry, Oral and Craniofacial Sciences, King’s College University of London, London SE1 9RT, UK; ana.angelova@kcl.ac.uk; 3 Physiology Graduate Program, North Carolina State University, Raleigh, NC 27695, USA; pemozdzi@ncsu.edu; 4 Prestage Department of Poultry Science, North Carolina State University, Raleigh, NC 27695, USA; 5 Department of Anatomy, Poznan University of Medical Sciences, 60-781 Poznan, Poland; 6 Department of Diagnostics and Clinical Sciences, Institute of Veterinary Medicine, Nicolaus Copernicus University in Torun, 87-100 Torun, Poland; 7 Department of Biomaterials and Experimental Dentistry, Poznan University of Medical Sciences, Bukowska 70, 60-812 Poznan, Poland

**Keywords:** COVID-19, SARS-CoV-2, exosomes, extracellular vesicles

## Abstract

The COVID-19 pandemic is a global challenge, demanding researchers address different approaches in relation to prevention, diagnostics and therapeutics. Amongst the many tactics of tackling these therapeutic challenges, small extracellular vesicles (sEVs) or exosomes are emerging as a new frontier in the field of ameliorating viral infections. Exosomes are part of extracellular vesicles (EVs)—spherical biological structures with a lipid bilayer of a diameter of up to 5000 nm, which are released into the intercellular space by most types of eukaryotic cells, both in physiological and pathological states. EVs share structural similarities to viruses, such as small size, common mechanisms of biogenesis and mechanisms for cell entry. The role of EVs in promoting the viral spread by evading the immune response of the host, which is exhibited by retroviruses, indicates the potential for further investigation and possible manipulation of these processes when tackling the spread and treatment of COVID-19. The following paper introduces the topic of the use of exosomes in the treatment of viral infections, and presents the future prospects for the use of these EVs.

## 1. Brief History of Viral Pandemics

Viral pandemics are not a new phenomenon, as even the first steps towards transport and global development contributed to their formation and spread [1]. A pandemic, by definition, is an epidemic of a disease that occurs globally (as opposed to an epidemic that only covers specific areas) and typically affects a large proportion of the population. Despite the existence of many potential transmission possibilities, zoonotic infections account for a very large percentage of all such cases throughout human history [2]. Although it would seem that with the passage of time and limitation of human–animal interactions the risk of zoonotic infections decreases, this risk continues to occur as long as humans engage in hunting, animal-based food trade, exotic animal and pet trade or supporting wet markets [3]. The risk of zoonotic infection is influenced by the frequency of human-animal interactions, the species involved, and the nature of these interactions. Wolfe et al. proposed five stages of transmission of zoonotic pathogens [2]. The pathogen infects animals found only in natural conditions, then it evolves, leading to a risk of transmission to humans, but without the possibility of transmitting the pathogen from human to human. Next, it undergoes several cycles of secondary transmission between people. Furthermore, human–human infection takes place without the participation of animals. Finally, in the fifth stage, it begins to occur only in humans [2]. In recent years, concerns were raised among scientists that climate change also has a significant impact on the modulation of zoonotic infections in humans by enlarging the habitats of various zoonotic disease-carrying vectors [4,5]. The Russian influenza that occurred from 1889–1893 is an example of zoonotic viral infection, caused by the A/H3N8 virus [6]. Although the first mentions of an influenza pandemic appears as early as 1510 [7], it was the Russian flu that was the first, arguably reliably described and documented as an influenza pandemic [8]. Although the mortality rate in this pandemic was considered low, as case fatalities ranged from 0.10 to 0.28%, the virus—endemic in birds, horses and dogs—caused 1 million deaths within 3 years [8,9]. A quarter of a century later, the A/H1N1 virus, a result of the genetic adaptation of the avian flu virus to a new human host, caused another pandemic—the Spanish flu [10]. While infection rates were lower (25–33%) [11] than those of the Russian flu (60%) [9], it caused 50 million deaths worldwide between 1918 and 1919 [12]. The SARS-CoV virus, in 2003, similar in symptoms to the flu, originated in Guandong (China), with its reservoir most likely considered to be bats [13]. Due to the long incubation time and the relatively low infectivity, the virus only affected 29 countries [14]. Although its fatality rate was 9.7%, its features allowed for the efficient introduction of effective restrictions, and finally the reduction of fatality to 813 out of 8,437 reported cases [8]. Despite the aforementioned coronavirus epidemic, the world was not prepared for the SARS-CoV-2 pandemic, which started with the cases recorded for the first time in December 2019. As with SARS-CoV, the animal reservoir is likely bats [15], while the animal hosts are probably pangolins [16], not palm civets [17], as was the case with SARS-CoV. While in the majority (about 80%) of people infected with SARS-CoV-2 the disease is confined to the upper respiratory tract and is mild, in about 15% of patients, especially those over 65 years of age, the disease takes a severe form [18]. The asymptomatic incubation period (with or without a detectable viral count), a mild symptomatic period with a detectable virus, and finally the severe symptomatic stage with a detectable virus are the three phases into which this infection can be divided. These stages are the most reliable classification of disease progression, and have been compiled on the basis of clinical data from over 1000 patients [18,19,20]. The life cycle of SARS-CoV-2 in host cells begins with the S1 subunit of the Spike (S) protein, which binds to the cellular Angiotensin I converting enzyme 2 (ACE2) receptor. The six-helix bundle is formed by heptapeptide repeat 1 and 2 (HR1 and HR2), enabling fusion of the viral and cell membrane [21,22]. Upon entry of the virus into the host cell, the viral RNA is released into the cytoplasm, while the pp1a and pp1ab polyproteins are translated and cleaved. In this way, a replication transcription complex is formed that directs the production of negative sense RNA [(-) RNA]. An (-) RNA copy of the genome is produced during replication and used later as a template for the full-length (+) RNA genome. A subset of the subgenomic RNA encoding all the structural proteins is generated during transcription. The viral nucleocapsid consists of genomic RNA and N proteins in the cytoplasm and buds into the lumen of the ER-Golgi intermediate cavity (ERGIC). At the end of this process, virus particles are released from the infected cells by exocytosis [22]. Although the mechanism of SARS-CoV-2 seems to be quite well understood, its relapses in people who have already been infected are alarming. While studying re-infected patients, the source of the hidden SARS-COV-2 RNA remains unknown [23]. It has been speculated that one of the potential mechanisms of recurrence of COVID-19 infection may be the cellular transport pathway associated with the release of exosomes carrying SARS-CoV-2. It is possible that these extracellular vesicles, ranging in size from 20 to 140 nm, may play the role of a “Trojan horse” in viral RNA reappearance in cured COVID-19 patients [23].

## 2. Brief History of Exosomes

Exosomes were first described in 1981, as fragments of the membrane isolated from the biofluid [24]. The discovery of exosomes was made by confirming that the transferrin receptors in reticulocytes are associated with small vesicles, which are then secreted from the maturing reticulocytes into the extracellular space [25,26]. The term “exosome” was clarified a few years later by Rose Mamelak Johnstone, the head of the Department of Biochemistry at the Faculty of Medicine at McGill University in Canada [27]. In 1997, a structure called the “exosome complex” or, often wrongly, the human “exosome”, was additionally discovered [28]. These two concepts should not be confused, as the exosome complex (or PM/Scl complex) is a nucleolar macromolecular complex with ribonuclease properties [28]. Thus, it is important in mRNA degradation and ribosomal RNA processing [29]. Exosome complexes are found in both eukaryotic cells and archaea. In bacteria, however, there is a simpler complex with similar functions called the degradosome [30,31,32]. It is therefore incorrect to use the names “exosome” and “exosome complex” interchangeably. The initial confusion around exosomes contributed to a sharp expansion of interest among researchers, which continues to the present day. As a result, the following societies were created: The American Society for Exosomes, International Society for Extracellular Vesicles, and even a dedicated journal—Journal of Extracellular Vesicles. Unfortunately, as the deeper research on exosomes and similar structures is carried out, new challenges related to standardized nomenclature are noticed [33]. Exosomes are a subtype of extracellular vesicles—spherical biological structures released by most types of eukaryotic cells and distributed into the intercellular space in both physiological and pathological states. These particles are also incapable of replication due to the lack of a functional nucleus [34]. According to van der Pol et al., EV is an “umbrella term” for names such as argosomes, blebbing vesicles, budding vesicles dexosomes, ectosomes, exovesicles, extracellular membrane vesicles, matrix vesicles, membrane blebs, membrane particles, membrane vesicles, microparticles, microvesicles, nanovesicles, oncosomes, outer membrane blebs, outer membrane vesicles, prominosomes, prostasomes, shedding microvesicles, shedding vesicles, synaptic vesicles, texosomes and tolerosomes [35]. Such a multitude of interchangeably used names introduces great confusion to the nomenclature and causes frequent disputes among scientists. In response to the above issues, ISEV, in its updated guidelines from 2018, suggests standardizing not only the replacement names, but also the names of different EVs subtypes, differing from each other, inter alia, in size or place of origin [34].

As scientists have yet to come to a consensus on specific markers that would clearly distinguish, for example, exosomes from ectosomes, the authors of MISEV2018 strongly encourage the use of the general term “EVs” to describe all extracellular vesicles [34]. As we read in the guidelines, only in some cases (for example, when an exosome is caught in the act of release by live imaging techniques) are we able to confirm the origin of a given vesicle. “Therefore, other nomenclature rules are proposed, such as distinction due to (a) size—“small EV” (sEV) and “medium/large EV” (m/lEV), where the ranges are successively < 100 or < 200 nm for sEV and > 200 nm for m/lEV, (b) density (low, middle, high, with each range defined), (c) biochemical composition (e.g., CD63+/CD81+ EVs, Annexin A5-stained EVs), (d) prevailing conditions or the origin of the cells (e.g., apoptotic bodies, large oncosomes, hypoxic EVs, podocyte EVs) [34].

The first two of the above category proposals were also successfully implemented in the article by Joanna Kowal, one of the participants in a leading exosome research team led by Clothilde Théry [36].

Although recent guidelines suggest the use of the term EVs to denote a heterogeneous extracellular vesicle population, MISEV do not prohibit use of different terms to distinguish individual vesicles [33]. It is allowed as long as they are precisely defined [34]. The term “exosome” is defined as a small (< 200 nm) extracellular vesicle released on exocytosis of multi-vesicular bodies (MVBs) filled with intraluminal vesicles (ILVs) [34,37]. Nevertheless, the general term “small extracellular vesicles” in the title has been used in accordance with the latest MISEV guidelines.

## 3. Classification, Structure and Location of Exosomes

**Figure 1:**
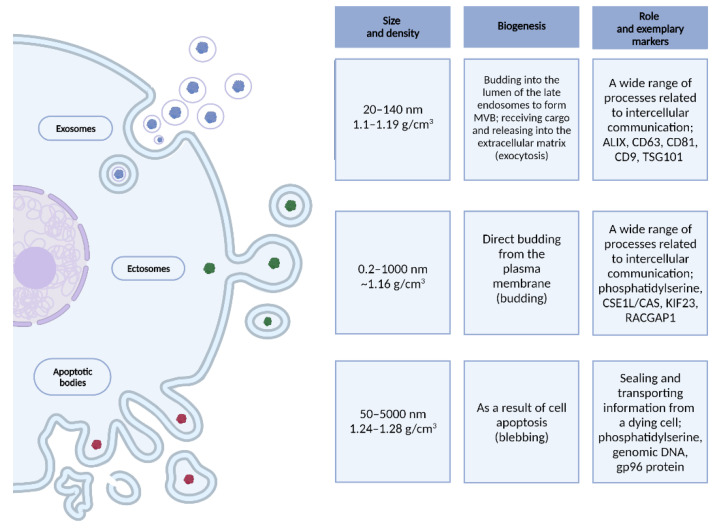
Size, density, biogenesis, role and exemplary markers of EVs–exosomes, ectosomes and apoptotic bodies. Created with BioRender.com.

Extracellular vesicles are spherical biological structures with a lipid bilayer of a diameter of up to 5000 nm, which are released into the intercellular space by most types of eukaryotic cells, both in physiological and pathological states [34,35,36,37,38,39]. The three best-known types of EVs are described in Figure 1. 

Although EVs are similar to each other, they most often differ in their place of origin. By studying exosomes, researchers found their most intense production mainly in dendritic cells, mast cells, B lymphocytes, adipocytes, neurons, and epithelial and endothelial cells [40]. An interesting finding was to prove that exosomes are also produced and secreted by cancer cells, in even greater numbers than by cells in physiological state [41]. Exosomes have been found in many body fluids: urine, blood, amniotic fluid, cerebrospinal fluid, saliva, breast milk, lymph, bile and ascites fluid, both in physiological and pathological states [42,43,44,45].

## 4. Formation, Secretion and Capture of Exosomes

**Figure 2:**
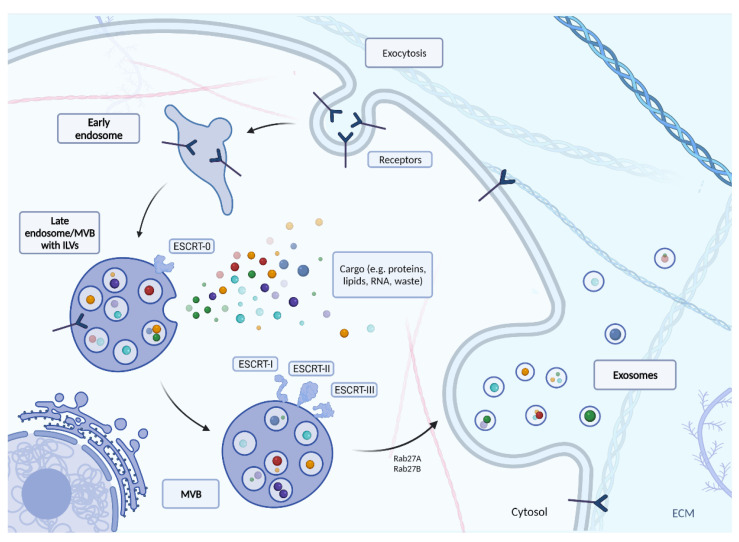
The biogenesis of exosomes with the use of endosomal sorting complex required for transport (ESCRT). Created with BioRender.com.

It is important to consider the lifecycle of an exosome to truly understand the role of exosomes in viral infection. The full process of creating exosomes via classic ESCRT path is described in Figure 2. In this mechanism, endocytosis initiates the production of exosomes. As a result, an early endosome is formed, which penetrates deeper into the cell and becomes the late endosome. During this phenomenon, its light begins to form intussusception, whose appearance results in the formation of internal vesicles called intraluminal vesicles (ILVs). These are precursors of exosomes, and at the time of formation, they receive their cargo (e.g., lipids, proteins, peptides, nucleic acids). The endosome-ILV complex is known as the multi-vesicular body (MVB), which begins to move towards the cell surface [46,47]. The MVB development process still requires ongoing research. Although several different mechanisms for MVB formation, vesicle release and vesicle sorting have been proposed, the best known is the endosomal sorting complex required for transport (ESCRT). The ESCRT mechanism mediates a pathway consisting of the ESCRT-0, -I, -II, -III protein complexes and associated ATPase Vps4, which converts chemical energy stored in the form of ATP into mechanical energy that breaks down ESCRT-III polymers). ESCRT 0 recognizes and retains ubiquitinated proteins until “packaged” into the membrane of the late endosome. ESCRT-I and-II recognize ESCRT-0 and begin to involute the membrane into the centre of MVB. The ESCRT-II then forms a spiral structure that tightens the area around the newly formed follicle to form its lumen. At the very end, a protein (ATPase VPS4) directs the invagination of the membrane, resulting in a cargo-filled vesicle [48].

There is also another way of exosome biogenesis in which ESCRT plays a role–the interaction of ESCRT-III/ALIX with syndecan (the transmembrane proteoglycan receptor) and syntenin, as the binding component [49,50]. Syndecans build up on the MVB membrane, resulting in a cleavage of the syndecan auto-repulsive domain. The binding of syntenin to the syndecan bundle is possible due to the concentration of syndecans on the membrane. The ESCRT-III unit is then recruited with VPS4 through interaction of syntenin with ALIX. This leads to the budding of the endosomal membrane inward and being abscissed. De-ubiquitination is finally initiated by ALIX and occurs prior to incorporation of proteins into ILV [49,51].

Tetraspanin-Mediated Biogenesis (TMB) and Lipid-Mediated Biogenesis (LMB), belonging to the ESCRT-independent pathways are additional pathways for consideration [49]. The tetraspanin protein family is known to have two extracellular and three intracellular regions. In the process of glycosylation, they form oligomers and a protein-enriched microdomain in the plasma membrane. Tetraspanin proteins can induce budding inward of the endosomal late membrane, thereby forming exosomes [52,53]. This is possible due to their conical conformation and the ability to merge into microdomains [54]. Tetraspanins are abundant in exosomes and could therefore be used as biomarkers for confirming the presence of exosomes [55,56]. The second ESCRT-independent mechanism is based on the content of ceramides, cholesterol and sphingolipids in exosomes. This pathway begins with the conversion of sphingomyelin to ceramide by neutral sphingomyelinase 2. Phospholipase D2 then converts phosphatidylcholine to phosphatidic acid (PA). The ceramide and PA generated in this way on the MVB limiting membrane form a conical structure that may contribute to the negative curvature of the endosomal membrane [57,58]. The result of this process is inward budding and the final formation of ILVs.

The transport of MVB to the cell membrane is mainly driven by the Rab family of small GTPase proteins. Rab family proteins also take part in the formation of vesicles inside the MVB, controlling their mobility by interacting with the cytoskeleton of the cell, and docking the vesicles to their destinations near the cell membrane ending with the release of vesicles into the intercellular matrix [40,59]. For example, Rab11 controls the release of TfR and Hsc70 containing exosomes in K562 cells (leukemic cells) [60]. Together with Rab35, Rab11 is necessary for the secretion of exosomes loaded with anthrax toxin in the human RPE1 cell line (retinal pigment epithelial cells) [61]. Finally, the participation of Rab27A and Rab27B in management of exosomes has been confirmed in: mouse melanoma, mouse breast cancer, and human squamous cell carcinoma cells for Rab27A [62,63,64] and in HeLa cells both for Rab27A and Rab27B [65]. Knockdown of *RAB27A* may result in a reduction in the secretion of exosomes to the extracellular space [66]. Rab27A also influences exosome release by controlling the fusion of amphisomes with the plasma membrane [67]. According to Ostrowski et al., the key role of Rab27A and Rab27B in exosome secretion was promoting multivesicular endosomes (MVE) targeting at the cell periphery and their docking in the plasma membrane [65]. Therefore, both *RAB27A* and *RAB27B* silenced cells produce half as many exosomes as unmodified cells [65]. Additionally, silencing *RAB27A* increases the size of the MVE, while silencing *RAB27B* redistributes the MVE towards the perinuclear region, instead of towards the plasma membrane [65].

Once the MVBs are near the cell surface, their membranes fuse with each other. Vesicles located inside the MVB are transported outside the cell, thus becoming exosomes [68]. Located in the extracellular matrix, exosomes are segregated and delegated to different places depending on their load. They can transmit information to acceptor cells without delivering their cargo to them, acting only on their surface. Membrane based interactions may happen during an immune response, where exosomes carry the major histocompatibility complex (MHC). The interactions create a set of proteins responsible for the presentation of antigens to T lymphocytes [69]. An exosome with such content only activates receptors on the surface of T lymphocytes, without introducing its cargo inside the lymphocytes [70]. After being released from the MVB, the exosome may also be directed to the lysosome for degradation or be “recycled” for further release inside cells [71]. In the case of breast cancer, this type of recycling makes it possible to associate Wnt11 with internalized CD81 derived from fibroblasts. Releasing exosomes, or “recycling,” enhances the viability and mobility of breast cancer cells through Wnt-related signaling [72].

However, the main feature of exosomes is their role as carrier molecules protected by a lipid bilayer. The role of exosomes in tumor progression has been previously described in the literature, for example through the exosome mediated exchange of RNA between glioblastoma and endothelial cells [73], oncogenic variant III epidermal growth factor receptor (EGFRyIII) between glioblastoma cells [74], and oncogenic DNA sequences and retrotransposon elements between medulloblastoma and endothelial cells [75]. Protein transport in the nervous system has also been reported, where through exosomes, the transfer of cytosolic synaptotagmine-4 (SYT4) from presynaptic to postsynaptic cells allowed for retrograde control of presynaptic activity [76]. Exosomes can also carry toxic components such as prion proteins (PrPSc) that are loaded into exosomes to camouflage and target specific cells in a physiological state [77].

**Figure 3:**
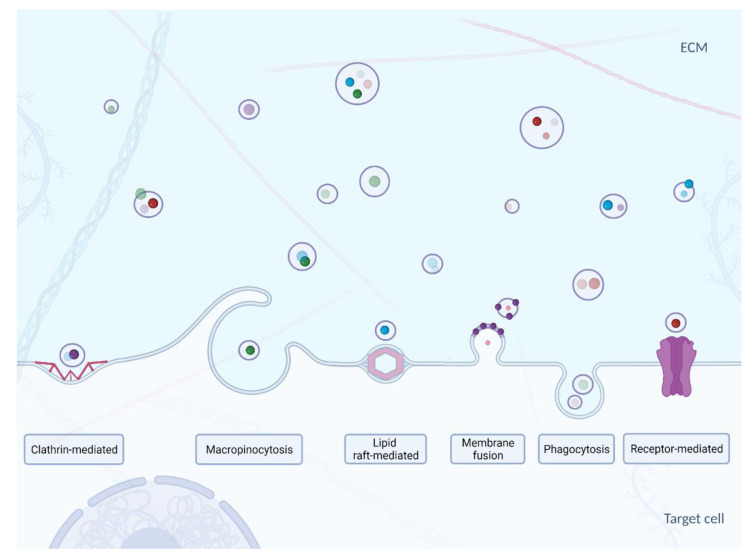
Uptake mechanisms of exosomes–clathrin-mediated [84], macropinocythosis [85], lipid raft-mediated [86], membrane fusion [87], phagocythosis [88] and receptor-mediated [89]. Created with BioRender.com.

The mechanisms of exosome uptake and delivery of their content to the cytosol of acceptor cells (Figure 3) have not yet been fully characterized. Nevertheless, it is known that the first step in exosome capture is “targeting” of the acceptor cell [78]. It has been suggested that the transfer of exosomal miRNAs may modulate the biological functions of the acceptor cells. However, indicating the difference between exogenous miRNA activity from the endogenous miRNA function did not occur so that the targeting process remains yet to be fully elucidated. Small EVs emanating from oligodendrocytes are preferentially internalized by microglial cells and not, as might be expected, by neurons [79]. Exosomes secreted by neurons in primary culture are only taken up by other neurons, while those from the neuroblastoma cell line bind just as well with astrocytes [80]. In contrast, HeLa cells are able to take up a wide variety of exosomes with different charges produced by different cells [81]. It was also found that exosome cargo transfer can also occur between the mouse and the human model [82]. CD47, an integrin-bound protein that protects cells from phagocytosis, and it is often found on the surface of the exosome. It has been shown to extend the circulation time of the exosomes in the blood, preventing its phagocytosis by macrophages and monocytes [83]. This led to more efficient targeting of exosomes delivering short interfering RNA to pancreatic cells, suggesting that targeting specific cells could also be achieved through an adverse selection mechanism [83].

The second step in exosome capture is selection of the point of entry into the acceptor cell. It is not entirely clear whether it is due to a non-specific process such as macropinocytosis or phagocytosis, or through a specific receptor-dependent pathway [90]. It has been suggested that proteins on the surface of EVs and acceptor cells are involved in the uptake of various EVs: lectins, integrins, proteoglycans, T-lymphocyte immunoglobulin, as well as the Tim4 protein, containing the mucin 4 domain [91,92,93]. Many types of EVs, including exosomes, share surface proteins, and it is possible that some of them act as a general ligand for the receptor, allowing the internalization (absorption) of vesicles, similar to the low-density lipoprotein uptake mechanism [94]. The internalization of EVs appears to occur through multiple pathways, which include endocytosis via both clathrin-dependent and non-clathrin mechanisms [90,95].

The third and final step in exosome internalization is delivering the contents to the receiving (acceptor) cell. In many studies, the final result of internalization is not deliberated or assessed by researchers, which makes it difficult to draw conclusions about the functional consequences for cargo transport via an exosome or other EV. The plasma membrane has been proposed as a target membrane for content delivery through membrane fusion with an exosome [96], which is the case for DNA-transferable EVs derived from the cell membrane, as their larger size may limit the success of internalization. Most publications now report that exosomes enter cells by internalization, prior to discharge of cargo. Thus, the endosome is the putative location for delivering the contents of the exosome. The topology of EVs (including exosomes) indicates that their fusion with the cytosol or endosome membranes in the cell would require specific machinery that would differ from the SNARE proteins used for intracellular vesicle fusion [78]. Other mechanisms that can control the release of EVs from endosomes cannot be ruled out, for example by releasing partially degraded material from ruptured endosomal or lysosomal compartments (endosomal and lysosomal compartments are temporary sorting stations at which the fate of the endosome or lysosome cargo is determined) [97]. Some unexpected release mechanisms have been described, including direct transfer to the nucleus or the endoplasmic reticulum through the contact of these compartments with the endosomes containing internalized EVs [98,99]. The observations may lead to directions for further analysis. 

## 5. Exosomes and Viruses

Over the last century, modern medicine has made significant advances in the prevention, diagnosis, and treatment of disease. However, inhibition of viral pathogens remains a significant challenge [100]. Implementation of an effective vaccine could be considered as the ultimate tool for stopping the pandemic, but the length and complex process of its development limits the application potential in the early stages of widespread infections. At the same time, the rapid mutation of the virus necessitates constant updates to the vaccine to maintain its efficacy [101]. Hence, there is a significant need for studies to understand the mechanisms and processes associated with the initial response of the organism when exposed to a virus. The new information could prevent the development of a disease, as well as facilitate its treatment.

Amongst the many approaches in tackling these challenges, extracellular vesicles (EVs) are emerging as the new frontier in prevention of viral infections. EVs are released into the intercellular space by most types of eukaryotic cells, both in physiological and pathological states, and thus must be taken into consideration while studying viral infections. Furthermore, EVs are involved in cell-to-cell communication by means of horizontal transfer of molecules at short and long distances [102].

EVs share structural similarities to viruses, such as: small size, common mechanisms of biogenesis and mechanisms for cell entry. Using the endocytic pathway, viruses enter the host cell and exit the cell by “budding” through the cell membrane [103]. The possible role of the EVs in incorporating pathogen derived materials and becoming delivery vectors was described by Gould et al. [104] as the “Trojan exosome hypothesis”. This intriguing role of EVs in promoting the viral spread by evading the immune response of the host, shown in retroviruses, indicates the potential for further investigation and possible manipulation of this process when tackling the spread and treatment of SARS-CoV-2 virus caused disease, known as COVID19.

**Figure 4:**
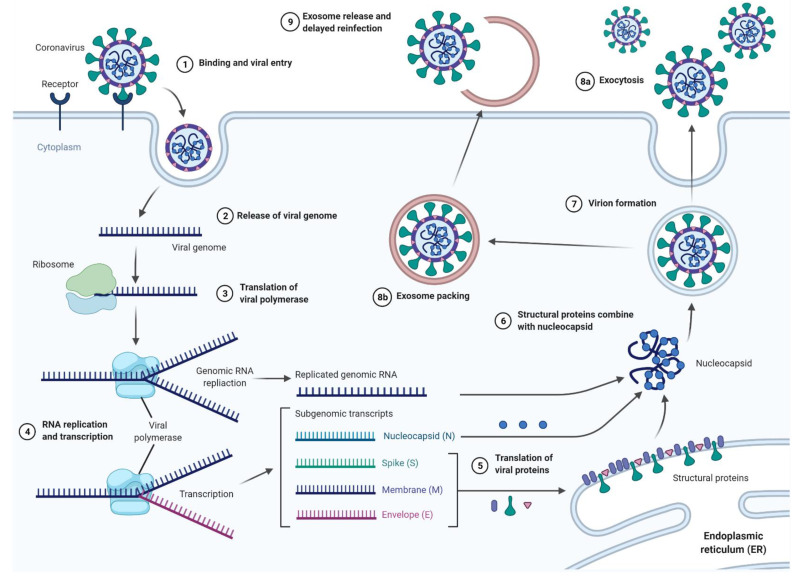
The process of SARS-CoV-2 virus infection, including the proposed mechanisms for exosome-based delayed reinfection. Created with BioRender.com.

**Figure 5:**
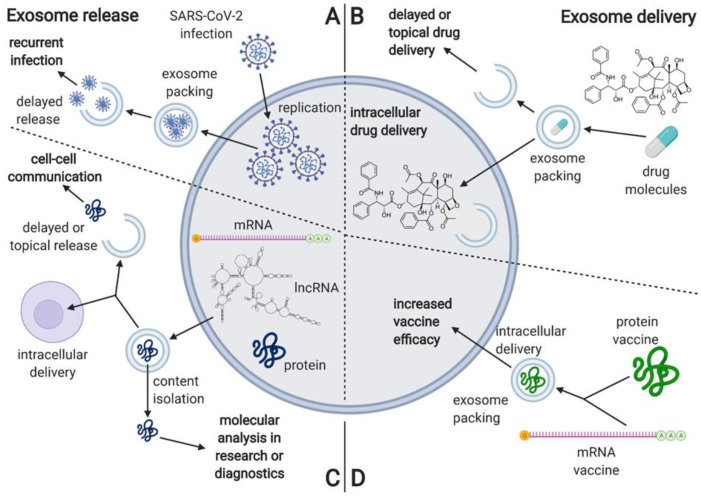
The characterization of processes associated with exosome release, including the proposed mechanism of delayed viral re-infection, as well as the proposed applications associated with exosome delivery. (**A**) he proposed mechanism of exosome involvement in SARS-CoV-2 reinfection; (**B**) overview of the proposed applications including exosome delivery; (**C**) the usual mechanisms and applications of exosome release; (**D**) overview of the potential applications of exosomes in vaccine delivery. Created with BioRender.com.

Furthermore, as exosomes and extracellular vesicles are potential mediators of viral infection, further studies are needed to explore their potential role in reinfection and reactivation. Some authors suggested the role of released SARS-CoV-2-loaded exosomes and other extracellular vesicles as potential mechanisms for the relapse of the COVID-19 infection. Extracellular vesicles containing Covid-19 may be a possible mechanism for the reappearance of the viral RNA in the recovered COVID-19 patients 7–14 days post discharge, suggesting that viral material was hidden within such exosomes or extracellular vesicles during this “silence” time period and then re-spread [23] (Figure 4 and Figure 5).

### Mapping Cellular “Gatekeepers” and Potential Usage of Exosomes

While researchers are racing to identify and map the details of the molecular infection profile of SARS-CoV-2 through proteomics at different times after infection [105], other groups are focusing on immunity as hosts, characterizing and highlighting the possible role of “cellular gatekeepers”. A recent study identified nerve- and airway-associated macrophages (NAMs), characterized as a distinct cell population from other lung resident macrophage subsets [106]. These cells were shown to highly express immunoregulatory genes under steady state and inflammatory conditions [107], indicating that alveolar macrophage (AM) derived exosomes modulate severity and outcome of acute lung injury. It was shown that macrophages were the major secretors for early secreted pro-inflammatory cytokines in the bronchoalveolar lavage fluid (BALF)-exosomes, which likely activated neutrophils to produce a variety of pro-inflammatory cytokines and IL-10. In turn, IL-10 in BALF-exosomes likely polarized macrophages to M2c, which may result in post-acute lung injury fibrosis, often seen in post-COVID19 cases [107].

The action of EVs is mostly associated with their content, dependent on the state of homeostasis, as well as the cells of origin, which can include molecules such as nucleic acids or proteins [108]. Hence, miRNAs and lncRNAs extracted from exosomes could serve as biomarkers and potential drug carriers/delivery vehicles and act as regulators of innate and acquired immunity through the stimulation of cytokine production, inflammatory responses, and antigen presentation, which opens the possibility of their potential application in therapies counteracting the harmful effects of viral infection [109].

After the outbreak of Sars-associated (SARS-CoV) in 2002, Kaute et al. 2007 [110] explored exosome-based vaccines containing the spike (S) proteins of SARS-CoV. The immunogenicity and efficacy of the S-containing exosomes were tested in mice and compared to vaccine delivering the S protein using adenoviral vector, as the most common approach in vaccine design,). Both S-containing exosomes and the adenoviral vector vaccine induced neutralizing antibody (Ab) production. After priming with the SARS-S exosomal vaccine and boosting with the adenoviral vector, the neutralizing antibody counts exceeded those observed in the convalescent serum of a SARS patient [110].

While most of the candidate and approved vaccines for SARS-CoV-2 are based on immunization with the viral spike protein delivered on viral vectors, encoded by injected mRNAs, or as purified protein, emerging publications propose exosomes to deliver mRNAs that encode antigens from multiple SARS-CoV-2 structural proteins [111]. Exosome mediated mRNA delivery for SARS-COV-2 vaccine is based on using purified exosomes that are loaded with mRNAs designed to express (i) an artificial fusion protein (LSNME), that contains portions of the viral spike, nucleocapsid, membrane, and envelope proteins; and (ii) a functional form of spike, establishing that this vaccine induced broad immunity to multiple SARS-CoV-2 proteins [111]. An interesting way of administering exosomes is through the nasal passages. Such a form of vaccine application is well known in the case of preventing influenza. In the case of SARS-CoV-2 vaccine, exosomes or other nanocarriers could be used for presenting the gene sequence of S protein.

Intranasal administration of vaccines would resemble the actual CoV-2 infection and could induce systemic immunity, and at the same time promote immunity of the mucosal barriers [112].

Furthermore, exploring the possibility of the regulation of systemic inflammatory response against the virus with the initial phases of the mucosal reaction at its entry point could improve the longevity of the acquired immunity.

The constant challenge in intranasal vaccination is to ensure safety while securing immunogenicity and subsequently sterilizing immunity. Intranasal administration of vaccines requires efficient adjuvants. Exosomes isolated from proinflammatory immune cells could be used to reinforce the immune response of a vaccine [113].

Exosomes can be efficient vehicles for the delivery of functional mRNAs for use as therapeutics. Furthermore, the implementation of mesenchymal stem cells (MSCs) and their exosomes (MSC-Exo) [114] may be one of the frontiers for further research aiming to design new vaccines and therapeutics to conquer the viruses that continue to put the world at risk.

## 6. Exosomes as Therapeutics in Viral Infections

Many types of nanoparticle structures have found their application in medicine, becoming a part of drug delivery systems. While liposomes are now at the forefront of virus and vaccine issues, cells are believed to offer other structures that can outperform traditional systems [115,116]. Meanwhile, Jesus et al. found that EVs isolated from lipopolysaccharide stimulated monocytes induced an immune response and thus the release of several cytokines inducing Th1 response [117]. It appears that the immunomodulatory effect of EV in cells and emphasized the potential of EVs as adjuvants in vaccine preparations [117,118]. Exosomes are now becoming a frequently suggested solution as cell-free alternatives for tissue regeneration and disease treatment, as they are able to deliver a therapeutic agent to a target in the organism without causing cellular toxicity or immune rejection [115,119]. Exosomes isolated from stem cells deserve special attention, given their immense regenerative potential [120]. Popowski et al. suggests that exosomes could be an excellent treatment option for SARS-CoV-2, dividing them into three categories: (1) natural exosomes, (2) ACE2 receptor presenting engineered exosomes as a binding “nanodecoy” and (3) engineered exosomes containing antiviral drugs for potential therapeutic approaches [115]. The available literature suggests that lung-derived exosomes would be better suited for the treatment of SARS-CoV-2 than exosomes derived from stem cells of other areas, despite their significant regenerative potential [121]. Natural exosomes isolated from these organs would prevent the multi-organ damage as well as the systemic cytokine storm, both of which occur when suffering from COVID-19 [122]. “Nanodecoys”, representing category II, would prevent the entry of viruses into the host cells by binding and tagging them for final immune cell elimination [123,124]. This strategy may neutralize host inflammation occurring with viral infections. To optimize exosome “nanodecoys”, mechanistic similarities between viruses and exosomes must be emphasized and applied [115,124]. The third and last category included antiviral drugs encapsulated in lung-delivered exosomes. As mentioned above, this strategy would not only be immunologically neutral, but would also reduce the danger of drug delivery beyond the target area [115]. The anti-inflammatory effects of exosomes may allow for better suppression of viral replication and suppression of complications such as acute respiratory distress syndrome (ARDS), often occurring when suffering from COVID-19 [115,125]. While COVID-19 is by far one of the most important issues today, the dangers of other viruses should not be overlooked. It has been recently stated that airway-derived exosomes can neutralize influenza virus and block infection of target cells [126]. Previously conducted studies have shown that respiratory epithelial cell lines-derived exosomes expressed sialylated mucins—a well-known receptor for influenza virus [127]. The airway-derived exosomes examined by Bedford et al. expressed sialic acid receptors on its surface suggesting that sialic acid receptors expressed on the surface of airway-derived exosomes enable them to neutralize influenza virus [126]. The potential of exosomes to treat viral infections is no longer only being recognized, but also finally being implemented into more advanced research.

## 7. Conclusions and Future Perspective

Despite the ongoing research on exosomes in the context of viral infections, these structures still surprise with the range of their possible application. However, scientists should be aware that few biological tools are flawless, and although there have been suggested positive effects of EVs in the fight against viruses, exosomes can also promote inflammation [84]. Further development aims to provide expected therapeutic functions and clinical potential of exosomes considering their tissue origin and their targeting function. There are many reasons for the future innovative prophylactic and therapeutic approaches with the use of multifunctional biological nanoparticles. In the case of new, highly contagious and rapidly progressing diseases (such as COVID-19) it is important to seek solutions at all possible levels, taking into account new emerging opportunities, without compromising the quality of therapy [128].

## Data Availability

Not applicable.
